# Novel strategy for activating gene expression through triplex DNA formation targeting epigenetically suppressed genes†

**DOI:** 10.1039/d4cb00134f

**Published:** 2024-07-31

**Authors:** Ryotaro Notomi, Shigeki Sasaki, Yosuke Taniguchi

**Affiliations:** a Faculty of Medicine, Dentistry and Pharmaceutical Sciences, Okayama University 1-1-1 Tsushima-naka Kita-ku Okayama 700-8530 Japan y-taniguchi@okayama-u.ac.jp; b Graduate School of Pharmaceutical Sciences, Kyushu University 3-1-1 Maidashi Higashi-ku Fukuoka 812-8582 Japan; c Graduate School of Pharmaceutical Sciences, Nagasaki International University 22825-7 Huis Ten Bosch Machi Sasebo city Nagasaki 859-3298 Japan

## Abstract

Triplex DNA formation is a useful genomic targeting tool that is expected to have a wide range of applications, including the antigene method; however, there are fundamental limitations in its forming sequence. We recently extended the triplex DNA-forming sequence to methylated DNA sequences containing ^5m^CG base pairs by developing guanidino-dN, which is capable of recognizing a ^5m^CG base pair with high affinity. We herein investigated the effect of triplex DNA formation using TFOs with guanidino-dN on methylated DNA sequences at the promoter of the RASSF1A gene, whose expression is epigenetically suppressed by DNA methylation in MCF-7 cells, on gene expression. Interestingly, triplex DNA formation increased the expression of the RASSF1A gene at the transcript and protein levels. Furthermore, RASSF1A-activated MCF-7 cells exhibited cell growth suppressing activity. Changes in the expression of various genes associated with the promotion of apoptosis and breast cancer survival accompanied the activation of RASSF1A in cells exhibited antiproliferative activity. These results suggest the potential of increases in gene expression through triplex DNA formation as a new genomic targeting tool.

## Abstract 2

Gene transcription is activated by forming triplex DNA on the methylated dC-containing promoter sequence of the gene whose transcription was suppressed.

## Graphical abstract

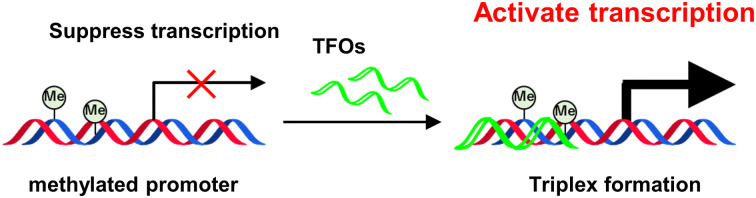

## Introduction

Triplex DNA is a higher-order structure formed by Triplex-forming oligonucleotides (TFOs) binding to duplex DNA. Triplex DNA formation using TFOs has potential as a useful genomic targeting tool because it may directly target duplex DNA with high sequence specificity. The most actively studied method is the antigene method, which inhibits the transcription process in gene expression by inhibiting the approach of transcription factors and RNA polymerases to the target sequence through the formation of triplex DNA.^1–3^ Wang *et al.* demonstrated antiproliferative activity in some cancer cells through reducing the transcription of taurine upregulated gene 1 (TUG1), vascular endothelial growth factor A (VEGFA), high mobility group AT-hook (HMGA) and MET proto-oncogene, receptor tyrosine kinase (c-MET) related with various cancers by forming triplex DNA using TFOs containing an artificial nucleoside analogue, ^3Me^AP-ΨdC, targeting these genes.^4^ Triplex DNA has also been examined using other approaches. McGorman *et al.* used artificial metallo-nuclease (AMN)-conjugated TFOs to form triplex DNA at the target site, resulting in target-specific DNA enzymatic cleavage and replication inhibition.^5^ Chen *et al.* enabled target sequence-specific fluorescence detection in PCR products by the formation of triplex DNA with TFOs containing perylene derivatives.^6^ Therefore, the development of various applications using triplex DNA formation is expected.

**Fig. 1:**
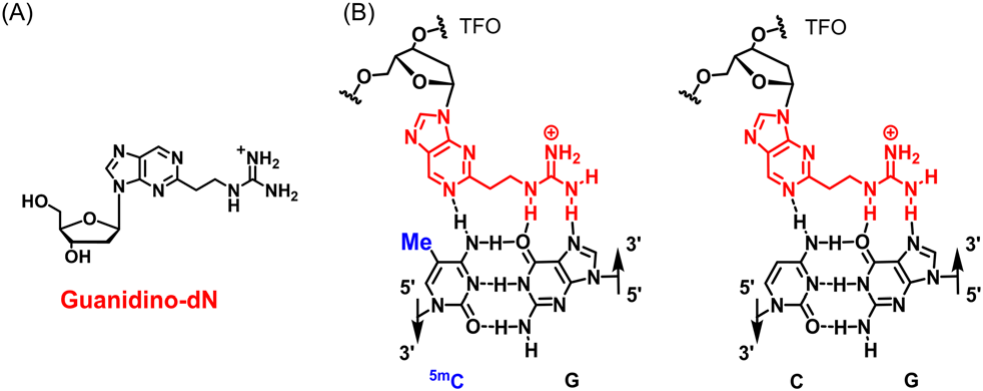
(A) Structure of guanidino-dN. (B) Predicted recognition style of guanidino-dN, which recognizes ^5m^CG (left) and CG (right) base pairs.

However, there is a fundamental limitation in the formation of triplex DNA. In triplex DNA, the natural nucleosides of TFOs, such as guanine (G), adenine (A), and thymine (T), recognize the GC and AT base pairs of duplex DNA *via* revers Hoogsteen hydrogen bonds.^7,8^ However, there are no natural nucleosides that form stable hydrogen bonds to the CG and TA base pairs. Therefore, stable triplex DNA cannot be formed when these base pairs are present in the target sequence, and sequences that form triplex DNA are limited to polypurine–polypyrimidine sequences. Therefore, artificial nucleoside analogues that recognize these inversion sites have been actively developed.^9–13^ On the other hand, between 60 and 90% of cytosine (C) in cytosine–phosphate–guanine (CpG) in genomic DNA are methylated to 5-methylcytosine (^5m^C), which is involved in the epigenetic suppression of gene expression without sequence variations.^14^ This epigenetic regulation involving DNA methylation is an extremely important mechanism for the survival of organisms, and abnormalities in DNA methylation are associated with various diseases, including cancer.^15^ For example, the hypermethylation of the promoter of tumor protein p53, a known cancer suppressor gene, has been associated with various cancers, including liver and breast cancers, and is being actively studied.^16,17^ The hypermethylation of the cyclin-dependent kinase inhibitor 2A gene has also been associated with colorectal cancer and non-small cell lung cancer and is a target gene that has attracted much attention.^18^ The ^5m^CG base pair in these highly hypermethylated DNAs is also one of the inversion sites in triplex DNA formation, and natural nucleosides cannot form stable triplex DNA in genomic DNA containing ^5m^C. To overcome this limitation, we developed the artificial nucleoside analogue, guanidino-dN (Fig. 1A), which recognizes ^5m^CG and CG base pairs with high affinity in any sequence and achieved the expansion of triplex DNA formation to genomic sequences containing ^5m^CG base pairs (Fig. 1B).^19^

We herein investigated the effects of triplex DNA formation for the promoter of a targeting gene, the expression of which is suppressed by DNA methylation, to examine the potential for new applications of triplex DNA formation using TFOs.

## Results and discussion

**Scheme 1:**
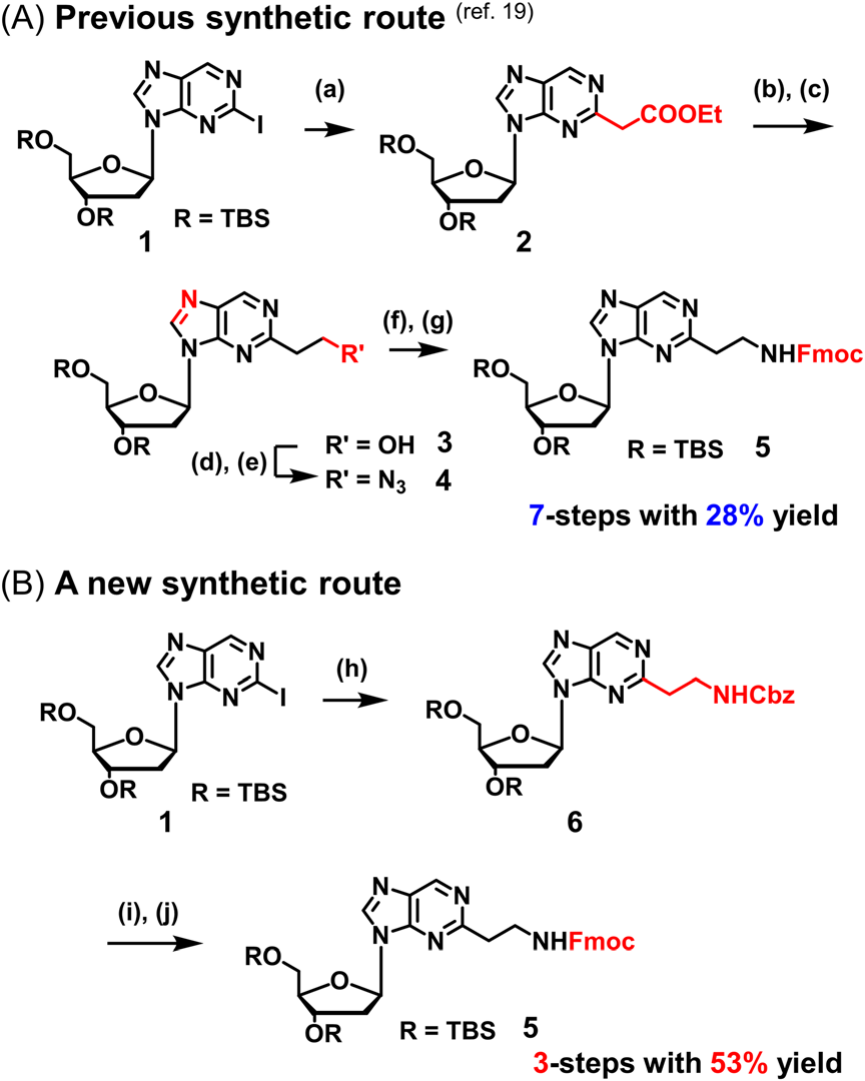
Optimization of the synthetic route of guanidino-dN. (A) Previous synthetic route, reagents, and conditions: (a) Pd(OAc)_2_, Xantphos, Cs_2_CO_3_, diethyl malonate, 135 °C, 87%, (b) LiAlH_4_, THF, -78 °C to 0 °C, (c) DDQ, DCM, r.t., 66% in 2 steps, (d) PPh_3_, CBr_4_, DCM, 0 °C to r.t., (e) NaN_3_, DMSO, r.t., 80% in 2 steps, (f) H_2_, 10% Pd/C, TEA, MeOH, r.t., (g) FmocCl, TEA, DCM, r.t., 69% in 2 steps. (B) A new synthetic route, reagents, and conditions: (h) benzyl-*N*-vinylcarbamate, 9-BBN, PdCl_2_(dppf), NaOH aq., THF, 65 °C, 65%, (i) Pd(OH)_2_/C, H_2_, MeOH, r.t., (j) FmocCl, TEA, DCM, r.t., 82% in 2 steps.

**Scheme 2:**
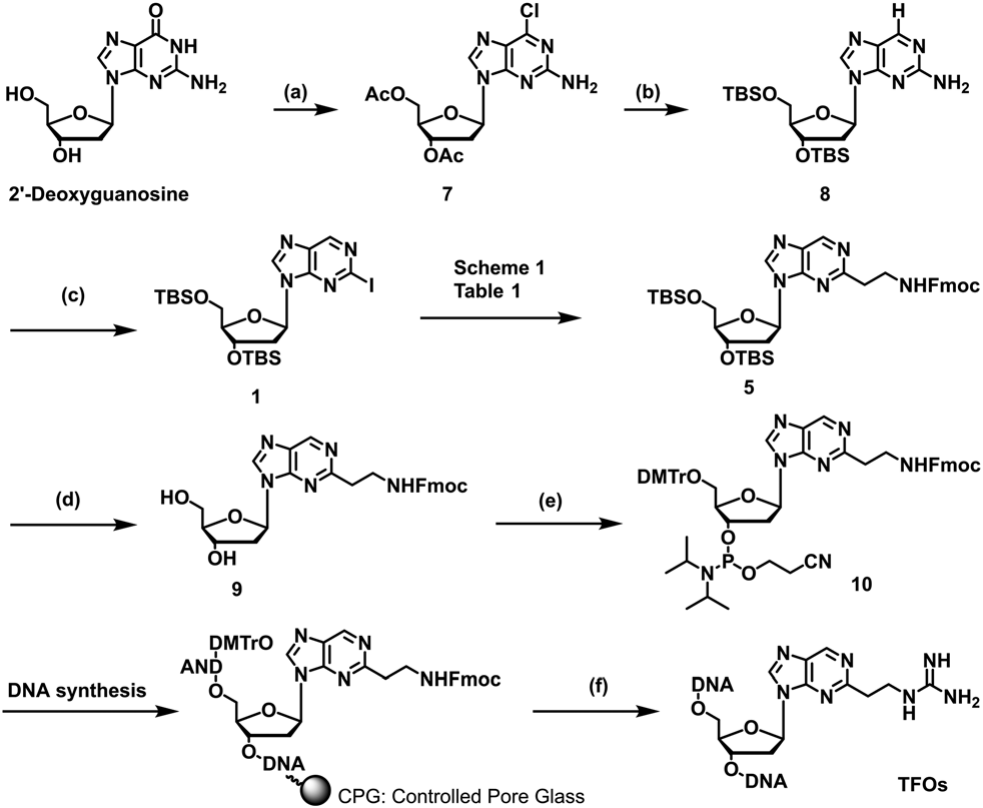
Synthesis of TFOs having guanidine-dN in this study. Reagents and conditions: (a) (1) acetic anhydride, DMAP, triethylamine, MeCN, r.t.; (2) POCl_3_, tetraethylammonium chloride, *N*,*N*-dimethylaniline, MeCN, 0 °C to 100 °C, 90% in 2 steps, (b) 10% Pd/C, H_2_, 28% NH_3_ aq., MeOH, r.t.; (2) TBSCl, Imidazole, DMF, r.t., 82% in 2 steps, (c) *tert*-butyl nitrite, CuI, CH_2_I_2_, MeCN, 70 °C, 49%; (d) NEt_3_-3HF, THF, r.t., quant., (e) (1) DMTrCl, pyridine, DCM, 75%; (2) 2-cyanoethyl-*N*,*N*-diisopropylchlorophosphoramidite, DIPEA, DCM, 0 °C, 77%, (f) (1) 28% NH_3_ aqueous solution at 55 °C; (2) 1-amidinopyrazole-HCl, TEA, MeOH, r.t.; (3) HPLC purification; (4) 5% AcOH aqueous solution.

We previously reported a scheme to synthesize guanidino-dN, which involved long steps and lacked good synthetic yield, making it difficult for biochemical applications.^19^ Therefore, we constructed a new simplified synthetic route that more easily provides guanidino-dN. In the previous route, we reported a 7-step reaction coupling with diethyl malonate to obtain 2, reduction of the ethyl ester by lithium aluminium hydride, reoxidation of the purine ring by 2,3-dichloro-5,6-dicyano-*p*-benzoquinone (DDQ) to obtain 3, bromination of the hydroxyl group using the Appel reaction, azidation using nucleophilic substitution to obtain 4, the reduction of azide by palladium on carbon, and the 9-fluorenylmethyloxycarbonyl (Fmoc) protection of an amino group to obtain 5 from compounds 1 to 5 with a total yield of 28% (Scheme 1A).^19^ In contrast, in our new simplified synthetic route, a olefin substrate was boronated by 9-borabicyclo [3.3.1] nonane (9-BBN) and linked directly to a purine ring of compound 1 using Suzuki–Miyaura cross coupling to give the compound 6. The conditions for coupling from compound 1 are shown in Table 1. Initially, coupling was performed using *N*-vinyacetamide as substrate and K_2_CO_3_ as base, but no progress in the reaction was observed (entry 1). Next, the formation of the target product was confirmed by using an excess of NaOH as a base, referring to the study by Roy *et al.* (entry 2).^20^ However, since the *N*-Ac protection was strong, the coupling was performed by changing the substrate to Benzyl-*N*-vinylcarbamate (entry 3). Furthermore, by adjusting the number of equivalents of the olefinic substrate and 9-BBN, the yield of the target product 6 was increased to 65% (entry 4). The benzyloxycarbonyl (Cbz) group of the amino group on the linker was deprotected by the reduction using Pearlman's catalyst and hydrogen gas, followed by Fmoc protection of the amino group, resulting in the synthesis of compound 5 in only 3 steps with a total yield of 53% (Scheme 1B). Finally, the synthesis of TFOs was successfully achieved from dG as a starting material by adopting the new steps developed in this study to the previously reported synthetic route (Scheme 2). In brief, the carbonyl group of dG was converted to a chloro group to give compound 7, which was reduced to give compound 8. After iodination of the amino group at 2-position, compound 5 was obtained using the method improved in this study. Then, it was converted to amidite compound 10, which was incorporated into DNA and guanidinated to obtain the desired TFOs (Tables S1 and S2, ESI†).

**Table 1:** Suzuki–Miyaura cross coupling reaction

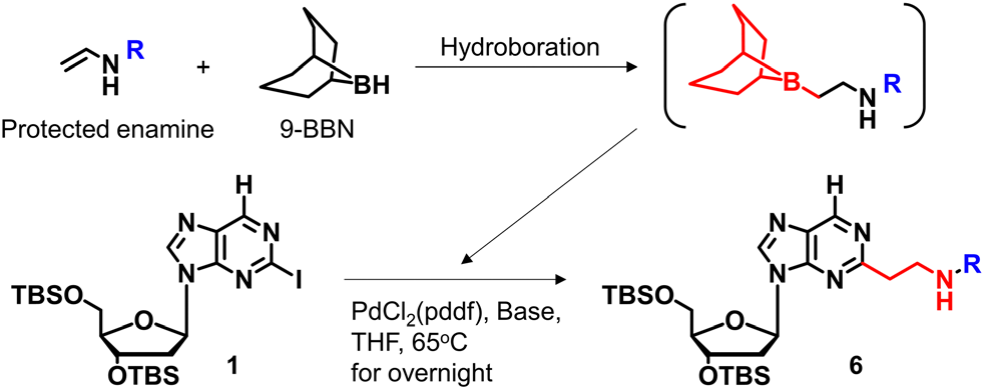					
Entry	Protected enamine (eq.)	9-BBN dimer (eq.)	Catalyst (0.1 eq.)	Base	Result (%)
1	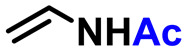	2.5	PdCl_2_(pddf)	K_2_CO_3_	Not proceeded
2	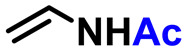	1.5	PdCl_2_(pddf)	NaOH aq.	34
3	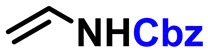	1.5	PdCl_2_(pddf)	NaOH aq.	41
4	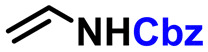	1.0	PdCl_2_(pddf)	NaOH aq.	65

**Fig. 2:**
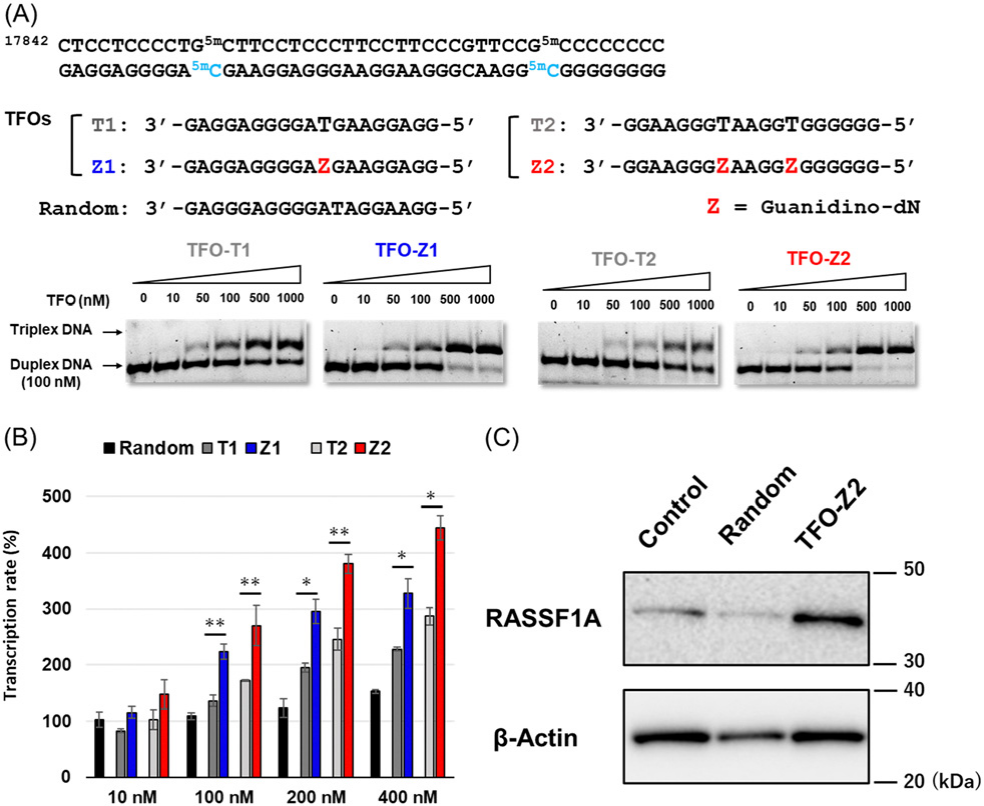
(A) The target sequence of the RASSF1A promoter and TFOs (TFO-T and TFO-Z) forming triplex DNA for the target sequence 1 and 2. FAM-labeled RASSF1A duplexes (25 bp; 100 nM) were incubated with increasing concentrations of each TFO (19-mer; 0–1000 nM) in buffer containing 20 mM Tris–HCl and 2.5 mM MgCl_2_ at 37 °C and pH 7.5. Electrophoresis was performed with a 10% non-denaturing polyacrylamide gel at 4 °C. (B) Activation of RASSF1A gene transcription in MCF-7 cells (2 × 10^4^ cells per well) transfected with TFOs (10–400 nM) and Lipofectamine 2000 for 3 h and incubated at 37 °C for 24 h. The transcription rate was calculated based on the ΔΔCt method by correcting the transcripts of RASSF1A normalized with that of GAPDH measured by qRT-PCR. (C) Activation of RASSF1A gene expression in MCF-7 cells transfected with TFOs. MCF-7 cells (8 × 10^4^ cells per well) were transfected with TFOs (random, TFO-Z2; 200 nM) and Lipofectamine 2000 for 3 h and then incubated at 37 °C for 24 h. The expression rate of RASSF1A was normalized with the expression of β-actin measured by western blotting.

Since the optimization of this synthetic route facilitated the supply of guanidino-dN, which recognizes ^5m^CG base pairs in triplex DNA, we attempted a new approach to target methylated DNA using triplex DNA formation. Ras association domain family member 1A (RASSF1A) is one of the representative tumor suppressor genes and its gene expression is suppressed by the hypermethylation of the promoter in some cancer cells.^21,22^ In the human breast cancer cell line MCF-7, the RASSF1A promoter is highly methylated^23,24^ and when its expression level was compared with that in HeLa cells with an unmethylated RASSF1A promoter^25^ by qRT-PCR, RASSF1A expression in MCF-7 was strongly suppressed (Fig. S1, ESI†). TFOs targeting promoter sequences 1 and 2 containing ^5m^C of this gene, TFO-Z1, Z2 and TFO-T1, T2, were designed such that their phosphate backbone was oriented antiparallel to the purine-rich DNA strand, and guanidino-dN (Z) or T was complementarily incorporated into the CG and ^5m^CG base pairs in the target sequences (Fig. 2A).^19^ The ability of these TFOs to form triplex DNA at the target sequences was confirmed by gel electrophoresis, and TFO-Z1 and Z2 containing the artificial nucleoside analogue formed more stable triplex DNA than the respective natural TFO-T1 and T2. Similar results were obtained when targeting unmethylated or methylated sequences in the RASSF1A promoter region (Fig. 2A or Fig. S2, respectively, ESI†).

Firstly, these TFOs and random TFO with rearranged bases of T1 were transfected into MCF-7 cells in which RASSF1A transcription was suppressed by the methylated promoter,^26^ and gene transcription was quantitatively analyzed by RT-PCR after 24 hours (Fig. 2B). RASSF1A transcript levels were corrected for glyceraldehyde-3-phosphate dehydrogenase (GAPDH) transcript levels and standardized with untreated control cells. The results obtained showed that random TFO did not significantly affect gene transcription, whereas TFO-T1 and T2 increased RASSF1A gene transcripts in a concentration-dependent manner. Furthermore, TFO-Z1 and Z2, incorporating artificial nucleoside analogue, increased RASSF1A gene transcription to a greater extent than their respective natural TFOs. The transcriptional activation of RASSF1A by TFO-Z2 showed an approximately 4.5-fold increase from that in untreated MCF-7 cells under 400 nM TFO conditions. This transcriptional activation of RASSF1A by TFO-Z was also observed in other cells containing hypermethylated promoters, such as HepG2 and A549 cells.^26,27^ Significant transcriptional activation of RASSF1A by TFO-Z2 was observed in HepG2, a human liver cancer cell (Fig. S3, ESI†), and slight activation by TFO-Z1 was observed in A549, a human lung cancer cell (Fig. S4, ESI†). On the other hand, this transcriptional activation was not observed for cells with the RASSF1A unmethylated promoters, such as HCT 116 and HeLa.^25,26^ HCT116, a human colon cancer cell, showed no significant activation by TFO-Z (Fig. S5, ESI†), while HeLa, a human cervical cancer cell, conversely suppressed RASSF1A transcription with high concentration TFO-Z2 (Fig. S6, ESI†). To further investigate this transcriptional activation by TFOs, we analyzed RASSF1A at the protein level using Western blotting (Fig. 2C). An analysis of RASSF1A protein levels corrected for β-actin showed that random TFO did not markedly affect RASSF1A protein levels, whereas cells transfected with TFO-Z2 showed significant increases in RASSF1A protein levels. TFO-Z2 induced an approximately 3-fold increase in RASSF1A protein levels, similar to the activation of RASSF1A gene transcription by TFO-Z2 (Fig. S7, ESI†). These results indicate that the activation of gene expression by TFO increased even downstream RASSF1A protein levels *via* the increased transcription of target genes.

**Fig. 3:**
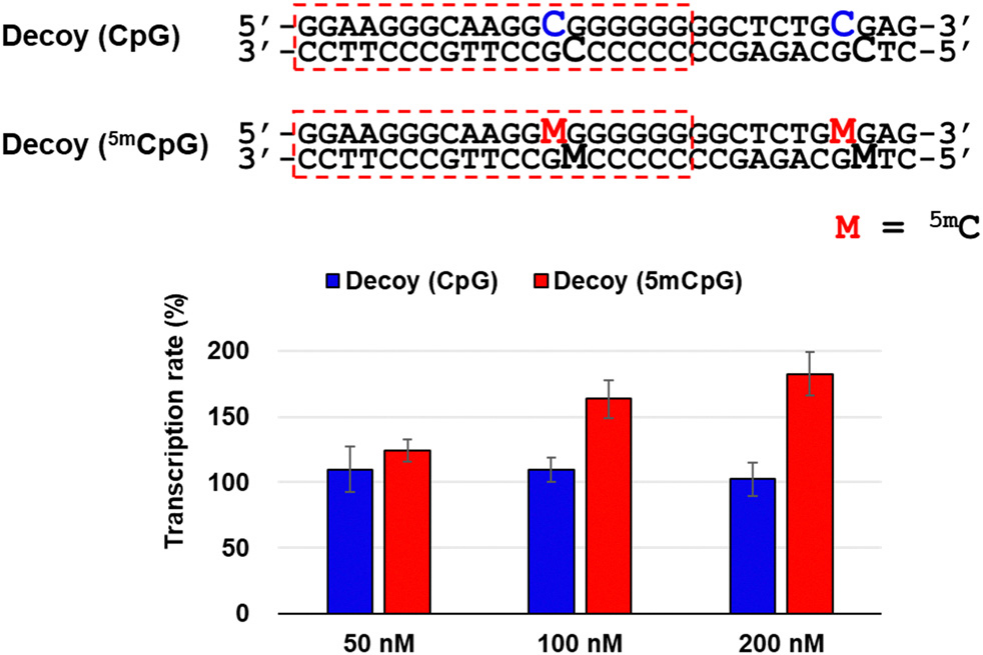
Effect for RASSF1A gene transcription in MCF-7 cells (2 × 10^4^ cells per well) transfected with Decoys containing CpG sites (50–200 nM) and Lipofectamine 2000 for 3 h and incubated at 37 °C for 24 h. The transcription rate was calculated based on the ΔΔCt method by correcting the transcripts of RASSF1A normalized with that of GAPDH measured by qRT-PCR.

To investigate the factors responsible for this transcriptional activation, we used decoy nucleic acids containing the target sequence of TFO-Z2 to study its effect on RASSF1A transcription (Fig. 3). No significant changes in transcription were observed in MCF-7 cells transfected with decoy (CpG). However, transfection of cells transfected with decoy (^5m^CpG), in which the C of the CpG site was changed to ^5m^C, activated RASSF1A transcription. This result suggests that the target sequence of TFOs may be acted upon by the methyl-CpG-binding domain (MBD) protein that recognizes ^5m^C, a protein that epigenetically suppresses gene expression.^28^ Therefore, TFOs may have prevented the suppression of gene expression by inhibiting MBD acting on the target sequence.

**Fig. 4:**
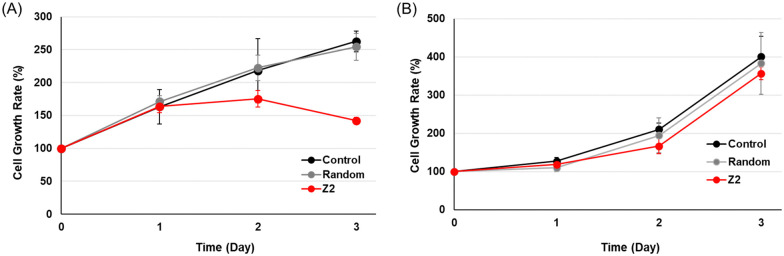
Anti-growth effects of cancer cells transfected with TFOs. (A) MCF-7 cells (5 × 10^3^ cells per well) or (B) HeLa cells (3 × 10^3^ cells per well) were transfected with TFOs (random, TFO-Z2; 200 nM) and Lipofectamine 2000 for 3 h and incubated at 37 °C for 1–3 days. CellTiter 96® AQueous One Solution Reagent was added, and cells were incubated at 37 °C for 1 h. Absorbance was measured at 490 nm.

The effects of the activation of the tumor suppressor gene RASSF1A by TFOs on cancer cell growth were examined using MTS fluorescence. MCF-7 or HeLa cells were transfected with 200 nM random TFO and TFO-Z2, and cell growth rates were plotted by culture times (Fig. 4). Under MCF-7 cell conditions, in which RASSF1A transcription was fully activated, cells transfected with random TFO showed similar growth to untreated cells, while those transfected with TFO-Z2 showed decreased cell growth on day 2 and further reductions on day 3. On the other hand, under HeLa cell conditions, which did not significantly affect RASSF1A transcription, cells transfected with random TFO and TFO-Z2 showed similar growth to untreated cells. This result may be attributed to an enhancement in the transcription of RASSF1A by TFO-Z2 in MCF-7 cells with a methylated promoter, followed by an increase in the downstream RASSF1A protein and its tumor suppressive function. The delayed the growth suppressing effect may be due to the time lag between the transcriptional activation of RNA by TFOs and the expression of protein function, which is a complex and long-term process.

**Fig. 5:**
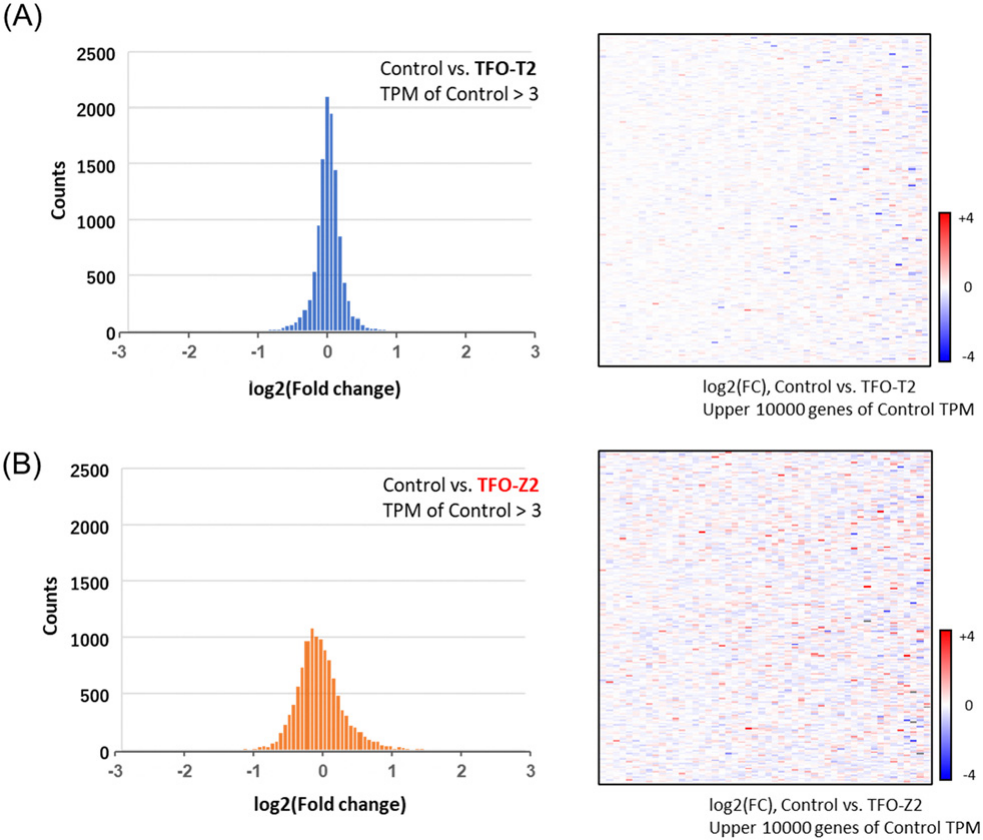
Histograms and heat maps of genes showing changes in their expression levels in MCF-7 cells transfected with TFOs (TFO-T2, -Z2; 200 nM) from those in the control. (A) The results for cells transfected with TFO-T2. (B) The results for cells transfected with TFO-Z2.

To search for factors that affect cancer cell growth, RNA sequencing was performed on MCF-7 cells transfected with TFOs in order to examine changes in the expression of a wide range of genes. Histograms and heat maps of the rate of change in gene expression in cells transfected with TFO-T2 or Z2 were generated and standardized against untreated controls (Fig. 5). Histograms and heat maps of MCF-7 cells transfected with TFO-T2 showed no significant changes in the expression levels of most genes (Fig. 5A). On the other hand, the results for TFO-Z2 transfected cells showed that the expression levels of many genes were significantly changed (Fig. 5B). These differences in the effects on gene expression in the cells may be due to the affinity of TFO for the target sequence.

**Fig. 6:**
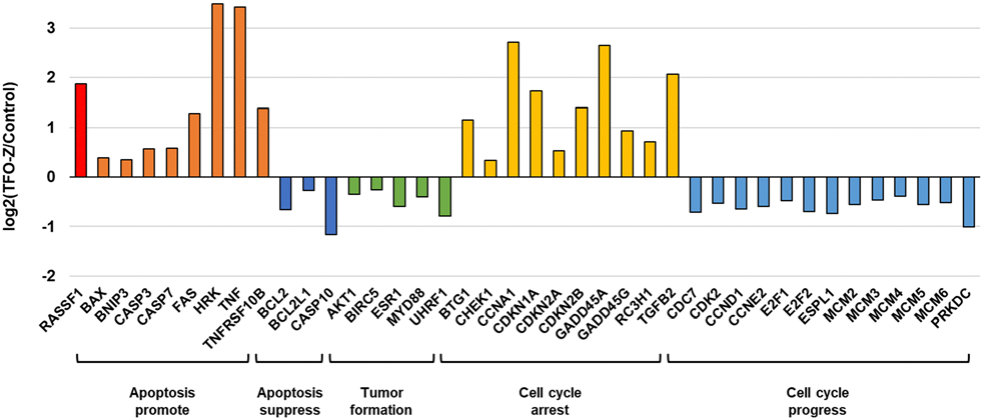
Genes whose expression was changed by TFO-Z2.

Furthermore, from the results of MCF-7 samples transfected with TFO-Z2, we extracted several genes and confirmed the fluctuations in their gene expression (Fig. 6). The results showed that many genes related to apoptosis and the cell cycle were altered, and that they were directed toward promoting apoptosis.^29–64^ Therefore, it is likely that the activation of RASSF1A by TFO caused changes in the expression of related genes and comprehensively suppressed the growth of MCF-7 cells.

Finally, we evaluated targeting cyclin dependent kinase inhibitor 2A (CDKN2A; p16) to see if transcriptional activation by TFO could be applied to other target genes. A sequence containing CpG sites were selected from the promoter sequence of the p16 gene, and TFO-T or Z containing T or Guanidino-dN at the complementary position of ^5m^CG base pairs were synthesized. The ability of these TFOs to form triplex DNA to the target sequence was evaluated by gel electrophoresis, and TFO-Z formed more stable triplex DNA (Fig. S8, ESI†). When these TFOs were transfected into HCT116 cells in which the p16 promoter was methylated,^65^ the p16 transcript increased in a TFO concentration-dependent manner, with TFO-Z showing a more substantial transcriptional activation effect (Fig. S9, ESI†). On the other hand, transfection of these TFOs into HepG2 cells with unmethylated promoters^66^ did not significantly increase transcripts (Fig. S10, ESI†). It was suggested that triplex DNA formation by TFO can act on target sequences in the methylated state and activate gene transcription, even for the p16 gene.

## Conclusions

We performed a novel biochemical approach through tri-plex DNA formation by optimizing the synthetic route of guanidino-dN, which recognizes ^5m^CG base pairs in triplex DNA. TFO-Z2 incorporating guanidino-dN, which forms stable triplex DNA with the RASSF1A promoter, exerted antigene effects at high concentrations in HeLa cells with an unmethylated promoter. In contrast, TFO-Z2 significantly activated the transcription of the RASSF1A gene in MCF-7 cells, in which the promoter was highly methylated and gene expression was epigenetically suppressed. The different effects on transcription by triplex DNA formation for sequences with different levels of methylation are very interesting, possibly due to differences in the mechanism of transcriptional inhibition by MBD, but further detailed investigation is needed. This gene activation was also observed at the protein level by Western blotting. MCF-7 cell growth was suppressed by the activation of the tumour suppressor gene RASSF1A, suggesting the involvement of changes in genes associated with apoptosis in breast cancer cells by RNA sequencing. Furthermore, this transcriptional activation of target genes by TFO has a similar effect on the p16 gene and may have a wide range of applications. This new approach, in which only single-stranded DNA, TFOs, affected various genes in cells and exhibited antiproliferative activity, may be an option for nucleic acid therapeutics in the future. We plan to extend the applicable sequences of triplex DNA formation to a wide range of genomic genes by developing new artificial nucleoside analogues.

## Author contributions

Y. T. designed the research, and R. N., S. S. and Y. T. wrote the paper. R. N. and Y. T. performed the experiments and analysed results. All authors discussed the results and commented on the manuscript.

## Data availability

Additional experimental data supporting this article are included in the ESI.† Reasonable requests for additional information can be made to the corresponding authors.

## Conflicts of interest

There are no conflicts to declare.

## Supplementary Material

CB-005-D4CB00134F-s001
