# Supplementary material for: Novel strategy for activating gene expression through triplex DNA formation targeting epigenetically suppressed genes
Source: RSC Chem Biol. 2024 Jul 31;5(9):884–90. doi: 10.1039/d4cb00134f (PMC11353075; doi:10.1039/d4cb00134f)
Supplement: CB-005-D4CB00134F-s001 [file CB-005-D4CB00134F-s001.pdf]

Supplementary Information (SI) for RSC Chemical Biology.
This journal is © The Royal Society of Chemistry 2024

Supporting information

Novel strategy for activating gene expression through triplex DNA formation targeting epigenetically suppressed genes

Ryotaro Notomi, Shigeki Sasaki, and Yosuke Taniguchi*

1. Oligonucleotides sequences used in this study (Table S1)..2

2. MALDI-TOF MS of TFOs used in this study (Table S2)..2

3. Experimental procedures 3

4. Experimental section: Synthesis of compound **6** and **5**..5

5. ^{1}H- and ^{13}C-NMR spectra of new compound **6**..6

6. Bar-graph of the transcription rate of RASSF1A gene (**Figure S1**)...................................7

7. The triplex-forming abilities (*K*s) of TFOs for RASSF1A promoter (**Figure S2**) 7

8. Activation of RASSF1A gene transcription in HepG2 cells (**Figure S3**)............................8

9. Activation of RASSF1A gene expression in A549 cells (**Figure S4**)..................................8

10. Effect of RASSF1A gene expression in HCT116 cells (**Figure S5**)..................................9

11. Effect of RASSF1A gene expression in HeLa cells (**Figure S6**)......................................9

12. Activation of RASSF1A gene expression in MCF-7 cells (**Figure S7**)............................10

13. The triplex-forming abilities (*K*s) of TFOs for p16 promoter (**Figure S8**).......................10

14. Activation of p16 gene expression in HCT116 cells (**Figure S9**)...................................11

15. Effect of p16 gene expression in HepG2 cells (**Figure S10**)..11

Table S1 Oligonucleotides sequences used in this study

Name	Sequence (5’ to 3’)
RASSF1A-Pu1 (^{5m}C)	GTGAGGAGGGGA^{5m}CGAAGGAGGGAAG
RASSF1A-Py1 (^{5m}C)	FAM-CTTCCCTCCTT^{5m}CGTCCCCTCCTCAC
RASSF1A-Pu2 (^{5m}C)	GAAGGAAGGGCAAGG^{5m}CGGGGGGGGC
RASSF1A-Py2 (^{5m}C)	FAM-GCCCCCCC^{5m}CGCCTTGCCCTTCCTTC
RASSF1A-Pu1(C)	GTGAGGAGGGGACGAAGGAGGGAAG
RASSF1A-Py1(C)	FAM-CTTCCCTCCTTCGTCCCCTCCTCAC
RASSF1A-Pu2(C)	GAAGGAAGGGCAAGGCGGGGGGGGC
RASSF1A-Py2(C)	FAM-GCCCCCCCCGCCTTGCCCTTCCTTC
p16-Pu1 (^{5m}C)	GAAGGAAA^{5m}CGGGG^{5m}CGGGGG^{5m}CGGATT
p16-Py1 (^{5m}C)	FAM-AATC^{5m}CGCCCC^{5m}CGCCC^{5m}CGTTTCCTTC
TFO-T1	GGAGGAAGTAGGGGAGGAG
TFO-T2	GGGGGGTGGAATGGGAAGG
TFO-Z1 (RASSF1A)	GGAGGAAGZAGGGGAGGAG (Z = guanidino-dN)
TFO-Z2 (RASSF1A)	GGGGGGZGGAAZGGGAAGG (Z = guanidino-dN)
Random TFO	GGAAGGATAGGGGAGGGAG
TFO-T (p16)	GGTGGGGGTGGGGTAAAGG (Z = guanidino-dN)
TFO-Z (p16)	GGZGGGGGZGGGGZAAAGG (Z = guanidino-dN)
RASSF1A-primer (forward)	CTTTTACCTGCCCAAGGATGC
RASSF1A-primer (reverse)	CACCTCCCCAGAGTCATTTTC
GAPDH-primer (forward)	GGAGTCCCTGCCACACTCA
GAPDH-primer (reverse)	GCCCCTCCCCTCTTCAAG
p16-primer (forward)	ATATGCCTTCCCCCACTACC
p16-primer (reverse)	CACATGAATGTGCGCTTAGG

Table S2 MALDI-TOF MS of TFOs used in this study

Name	Calculated $[M-H]^-$	Found
TFO-Z1 (RASSF1A)	6147.124	6145.465
TFO-Z2 (RASSF1A)	6235.188	6232.311
TFO-Z (p16)	6307.257	6304.826

Experimental procedures

General procedure for evaluating the triplex-forming ability of TFOs

FAM-labeled duplex DNA (100 nM) was incubated with increasing concentrations of TFOs (0–1000 nM) in buffer containing 20 mM Tris–HCl and 2.5 mM $MgCl_2$ at 37°C for 16 hrs and pH 7.5. Electrophoresis was performed at 4°C for 4 hrs using a 10% non-denatured polyacrylamide gel. Faster mobility bands were observed as unreacted duplex DNA, while slower mobility bands were detected as triplex DNA, which was a complex between duplex DNA and TFOs. The gel was visualized using the Luminoimage analyzer LAS-4000 (FUJIFILM), and the fluorescence intensity of each band was quantified for the calculation of association constants (*K*s). *K*s (10^6 M^{-1}) = [Triplex]/([TFO][Duplex]). The standard deviation (± S.D.) of each *K*s value was calculated from three independent experiments.

Cell Lines and culture

MCF-7, HeLa, HepG2, A549 and HCT116 were grown in the medium (DMEM, 10% FBS, 1% Penicillin-Streptomycin) at 37°C in a 5% CO_2 incubator.

TFOs transfection

MCF-7, HeLa, HepG2, A549 and HCT116 (2.0×10^4 cells/well, 500 μl medium) were seeded onto 24-well plate and grown in the medium at 37°C in a 5% CO_2 incubator. 24 hours later, the medium was removed and TFOs were transfected using Lipofectamine 2000 (Thermo Fischer) in Opti-MEM.

RNA extraction

After transfection, the medium was replaced and incubated for 24 hours. Total RNA was extracted from the cells using RNeasy Kits (QIAGEN).

Quantitative RT-PCR analysis

RNA was reverse transcribed to obtain cDNA using cDNA Synthesis Kit (Thermo Fischer). qPCR was performed using cDNA (10 ng/μl), RASSF1A primers (200 nM) and Luna Universal qPCR Master Mix (New England Biolabs). The obtained Ct values were normalized using the Ct values of GAPDH, and the RASSF1A transcription rate was calculated by the ΔΔCt method.

Western blotting

MCF-7 cells (8×10^4 cells/well in 6-well plate) were transfected with 200 nM TFOs and

Lipofectamine 2000 (Thermo Fischer) for 3 hrs and incubated at 37°C for 24 hrs. The cells were homogenized in SDS lysis buffer, quantified and lysates were separated on 10% SDS-polyacrylamide gels and transferred to PVDF membranes (Merck Millipore Ltd.) under 30V at 4°C overnight. After blocking with 5% skim milk, the membranes were probed with one of the following antibodies; anti-ACTB (Sigma-Aldrich, A2228) as a loading control (1:2000) and anti-RASSF1A (1:1000). This was followed by 1 hr incubation with secondary antibodies. The immunoreactive proteins were visualized.

MTS assay

HeLa (3.0×10^3 cells/well, 100 μl medium) or MCF-7 (5.0×10^3 cells/well, 100 μl medium) were seeded onto 96-well plate and grown in the medium at 37°C in a 5% CO_2 incubator. 24 hours later, the medium was removed and TFOs were transfected using Lipofectamine 2000 (Thermo Fischer) in Opti-MEM. After transfection, the medium was replaced and incubated for 1-3 days. For analysis of cell grown rate, the medium was added CellTiter 96®AQueous One Solution Reagent (Promega) and incubated at 37°C in a 5% CO_2 incubator for 1 hour. Then, absorbances at 490 nm of the mixture using 96-well plate reader.

RNA sequence

Total RNA samples were quantified and qualified by NanoDrop, Qubit RNA Assay (Thermo Fisher) and TapeStation RNA ScreenTape (Agilent). 210 ng of total RNA samples, which met the quality guideline, RIN: 8 or higher, were treated with NEBNext Poly(A) mRNA Magnetic Isolation Module (NEB) to enrich poly-A mRNA and to remove rRNA molecules. cDNA synthesis followed by transcriptome library preparation were conducted by NEBNext Ultra II Directional RNA Library Prep Kit for Illumina (NEB), where dUTP were incorporated in the process of the 2nd strand cDNA synthesis, instead of dTTP, that block PCR amplification against the 2nd strand templates. This enables to maintain the strandness of RNA transcripts. A 11-cycle PCR amplification was performed to increase library yield and also to incorporate sample barcodes into the library fragments. Resulting transcriptome libraries were quantified by Qubit DNA Assay (Thermo Fisher) and their fragment size distribution was estimated by TapeStation D1000 ScreenTape (Agilent). The libraries were then pooled/multiplexed at an equimolar amount each and loaded onto a next generation sequencing platform, NovaSeq 6000 (Illumina). Sequencing was carried out according to the manufacturer's instructions with 150 bp paired-end (PE) configuration, yielding approximately 20M PE reads per sample. Raw reads with low quality and adapter sequences were removed using Cutadapt 2.9. The remaining reads were mapped to the human genome (GRCh38 version 101) by HISAT2 v 2.2.0 (PMID: 25751142) and the gene expression was quantified using featureCounts v 2.6.0

(PMID: 24227677).

Experimental section

2’-Deoxy-3’,5’-*O*-(*tert*-butyldimethylsilyl)-2-*N*-(benzyloxycarbonyl)aminoethyl nebularine (6)

Under an argon atmosphere, a solution of Benzyl-*N*-vinylcarbamate (226 mg, 1.277 mmol) in THF (1.7 ml) was added 9-BBN dimer (208 mg, 0.852 mmol) at 0°C and stirred at room temperature for 6 hrs. The solution was added 3M NaOH aq. (1.7 ml) at 0°C and stirred at room temperature for 2 hrs. The reaction mixture was added to a solution of compound **1** (503 mg, 0.852 mmol) and $PdCl_2(dppf)\cdot CH_2Cl_2$ (69 mg, 0.085 mmol) in THF (5.1 ml) and stirred at 65°C for 12 hrs. The reaction mixture was added AcOEt (20 ml), washed with saturated aqueous $NaHCO_3$ (20 ml), dried over Na_2SO_4, concentrated under vacuum, and purified by column chromatography (SHOKO 10 g, Hexane/AcOEt = 100/0 to 40/60) to give a brown oil **6** (357 mg, 65%). ^{1}H-NMR (500 MHz, $CDCl_3$) σ (ppm) 8.93 (s, 1H), 8.39 (s, 1H), 7.25-7.30 (m, 5H), 6.44 (t, *J* = 6.25 Hz, 1H), 5.63 (bs, 1H), 5.01 (s, 2H), 4.56 (dd, *J* = 4.04, 9.28 Hz, 1H), 3.96 (dd, *J* = 3.40, 6.74 Hz, 1H), 3.82 (dd, *J* = 4.04, 11.41 Hz, 1H), 3.67-3.74 (m, 3H), 3.24 (t, *J* = 5.89 Hz, 2H), 2.52-2.59 (m, 1H), 2.37-2.43 (m, 1H), 0.84-0.86 (m, 18H), 0.02-0.04 (m, 12H); ^{13}C-NMR (125 MHz, $CDCl_3$) σ (ppm) 162.52, 156.37, 151.60, 144.23, 136.64, 132.75, 128.47, 128.42, 128.10, 128.03, 87.96, 84.19, 71.61, 66.55, 62.61, 41.42, 39.39, 38.36, 36.32, 31.57, 28.22, 25.93, 25.74, 22.64, 20.59, 18.41, 18.00, 14.11, -4.64, -4.80, -5.42, -5.49; ESI-HRMS (m/z): Calcd. for $C_{32}H_{51}N_5O_5Si_2Na$ $[M+Na]^+$: 664.3321, Found: 664.3307.

2’-Deoxy-3’,5’-*O*-(*tert*-butyldimethylsilyl)-2-*N*-(9-fluorenylmethyloxycarbonyl)amino ethylnebularine (5)

Under an argon atmosphere, a solution of compound **6** (357 mg, 0.556 mmol) in MeOH (5.6 ml) was added 20% palladium hydroxide on carbon (39 mg, 0.139 mmol), substituted by H_2 gas and stirred at room temperature for 19 hrs. The reaction mixture was celite filtered and concentrated under vacuum and dried in vacuo. Under an argon atmosphere, CH_2Cl_2 (5.1 ml) was added to the residue. The solution was added triethylamine (361 μl, 2.59 mmol) and 9-fluorenylmethyloxycarbonyl chloride (402 mg, 1.55 mmol) and stirred at room temperature for 30 minutes. The reaction mixture was concentrated under vacuum and purified by column chromatography (SHOKO 10 g, Hexane/AcOEt = 100/0 to 40/60) to give a brown oil **5** (333 mg, 82% in 2steps).

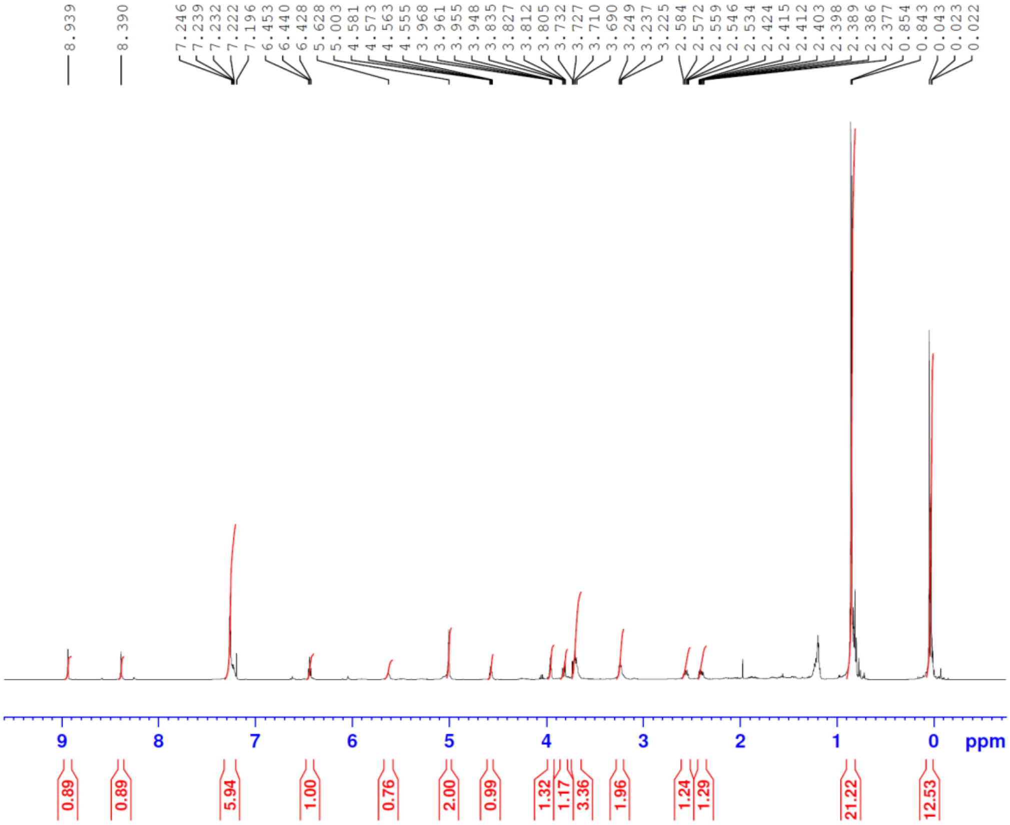
8.939
8.390
7.246
7.239
7.232
7.222
7.196
6.453
6.440
6.428
5.628
5.003
4.581
4.573
4.563
4.555
3.968
3.961
3.955
3.948
3.835
3.827
3.812
3.805
3.732
3.727
3.710
3.690
3.249
3.237
3.225
2.584
2.572
2.559
2.546
2.534
2.424
2.415
2.412
2.403
2.398
2.389
2.386
2.377
0.854
0.843
0.043
0.023
0.022
9 8 7 6 5 4 3 2 1 0 ppm
0.89
0.89
5.94
1.00
0.76
2.00
0.99
1.32
1.17
3.36
1.96
1.24
1.29
21.22
12.53

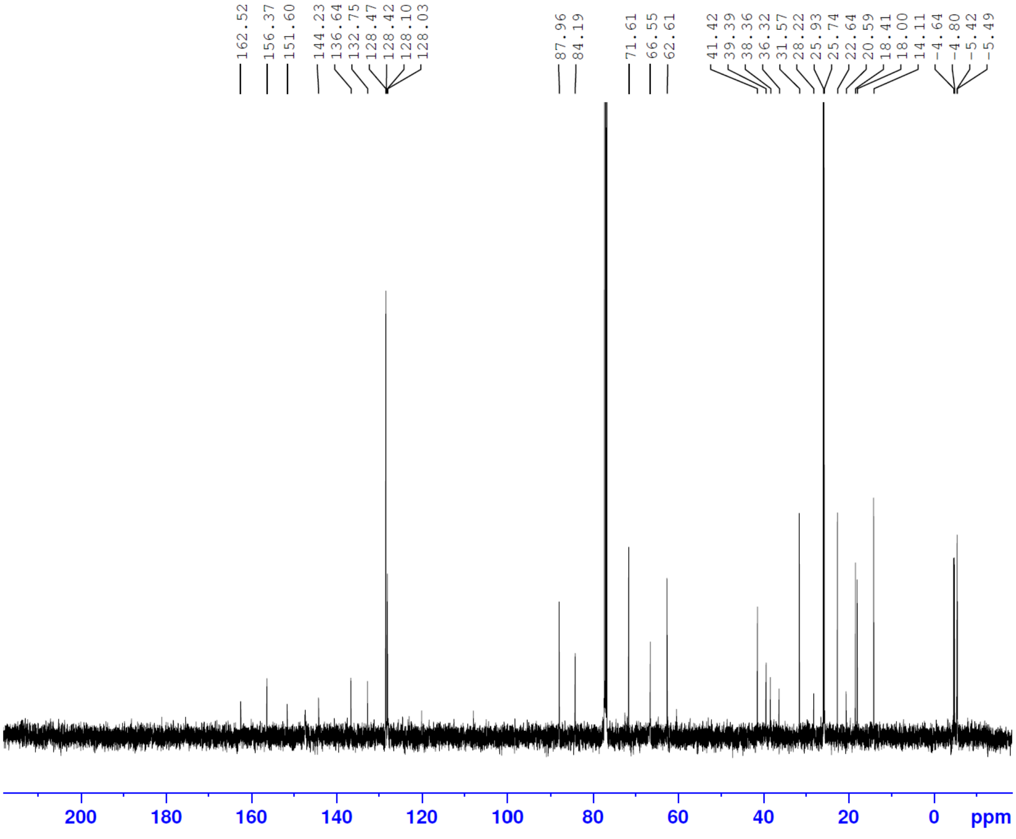
162.52
156.37
151.60
144.23
136.64
132.75
128.47
128.42
128.10
128.03
87.96
84.19
71.61
66.55
62.61
41.42
39.39
38.36
36.32
31.57
28.22
25.93
25.74
22.64
20.59
18.41
18.00
14.11
-4.64
-4.80
-5.42
-5.49
200 180 160 140 120 100 80 60 40 20 0 ppm

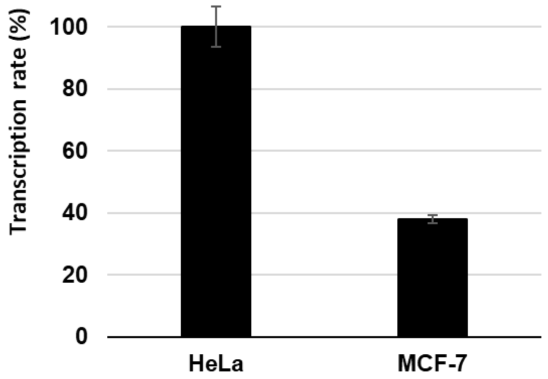

Figure S1 Bar-graph of the transcription rate of RASSF1A gene. HeLa or MCF-7 cells (2×10^4 cells/well in 24-well plate) were incubated at 37°C for 24 hrs. Real-time RT-PCR was performed using Luna® Universal qPCR Master Mix. Transcription rate of RASSF1A gene was calculated by *ΔΔ*Ct method with GAPDH gene. N = 3.

	Seq. 1	Seq. 2
TFO-T	4.54±0.92	0.75±0.44
**TFO-Z**	**8.44±1.88**	**14.5±1.51**

Figure S2 The triplex-forming abilities (*K*s) of TFOs (TFO-T1, T2, Z1 and Z2) for the target sequence in buffer containing 20 mM Tris-HCl and 2.5 mM $MgCl_2$ at 37°C and pH 7.5. Electrophoresis was performed with a 10% non-denaturing polyacrylamide gel at 4°C. *K*s (10^6 M^{-1}) = [Triplex] / ([TFO][Duplex])

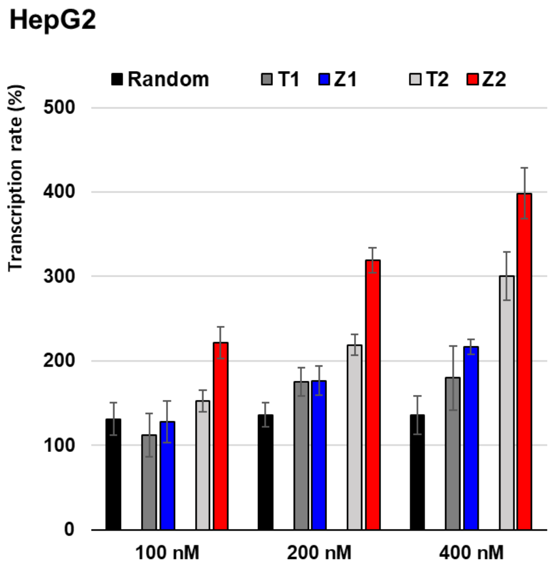

Figure S3 Activation of RASSF1A gene transcription in HepG2 cells (2×10^4 cells/well) transfected with TFOs (100-400 nM) and Lipofectamine 2000 for 3 hrs and incubated at 37°C for 24 hrs. The transcription rate was calculated based on the *ΔΔ*Ct method by correcting the transcripts of RASSF1A normalized with that of GAPDH measured by qRT-PCR.

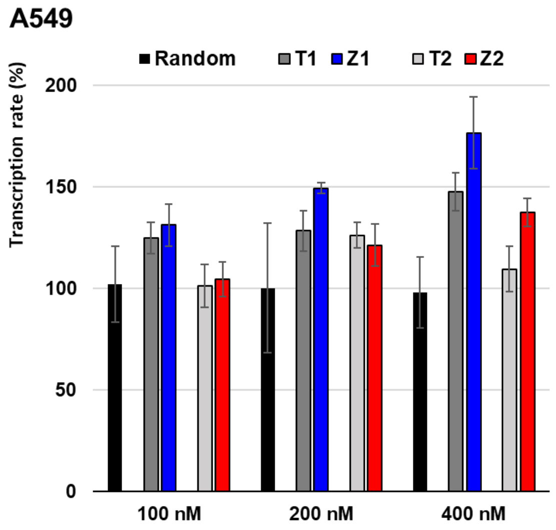

Figure S4 Activation of RASSF1A gene transcription in A549 cells (2×10^4 cells/well) transfected with TFOs (100-400 nM) and Lipofectamine 2000 for 3 hrs and incubated at 37°C for 24 hrs. The transcription rate was calculated based on the *ΔΔ*Ct method by correcting the transcripts of RASSF1A normalized with that of GAPDH measured by qRT-PCR.

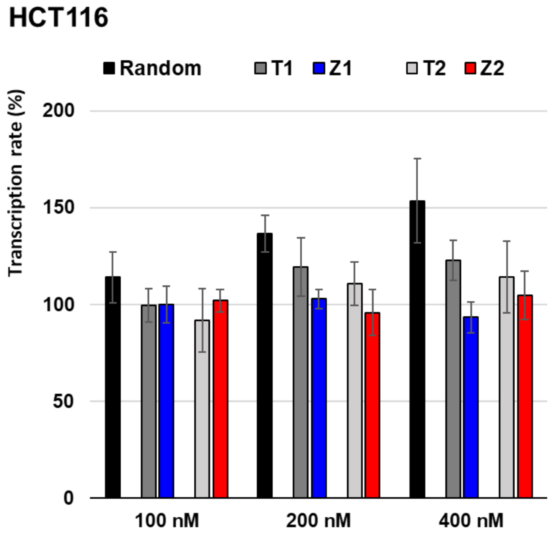

Figure S5 Effect of RASSF1A gene transcription in HCT116 cells ($2×10^4$ cells/well) transfected with TFOs (100-400 nM) and Lipofectamine 2000 for 6 hrs and incubated at 37°C for 24 hrs. The transcription rate was calculated based on the *ΔΔ*Ct method by correcting the transcripts of RASSF1A normalized with that of GAPDH measured by qRT-PCR.

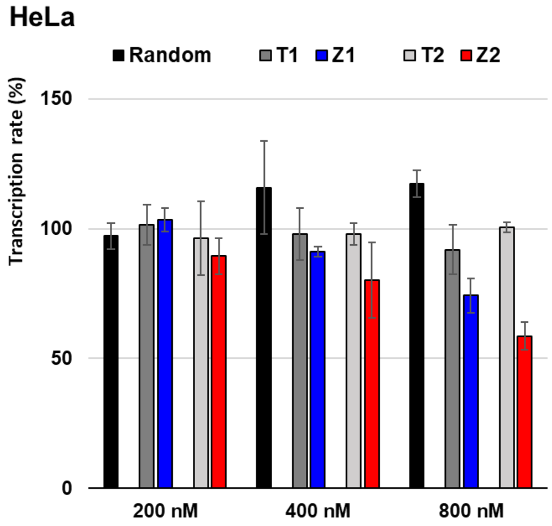

Figure S6 Effect of RASSF1A gene transcription in HeLa cells ($2×10^4$ cells/well) transfected with TFOs (200-800 nM) and Lipofectamine 2000 for 6 hrs and incubated at 37°C for 24 hrs. The transcription rate was calculated based on the *ΔΔ*Ct method by correcting the transcripts of RASSF1A normalized with that of GAPDH measured by qRT-PCR.

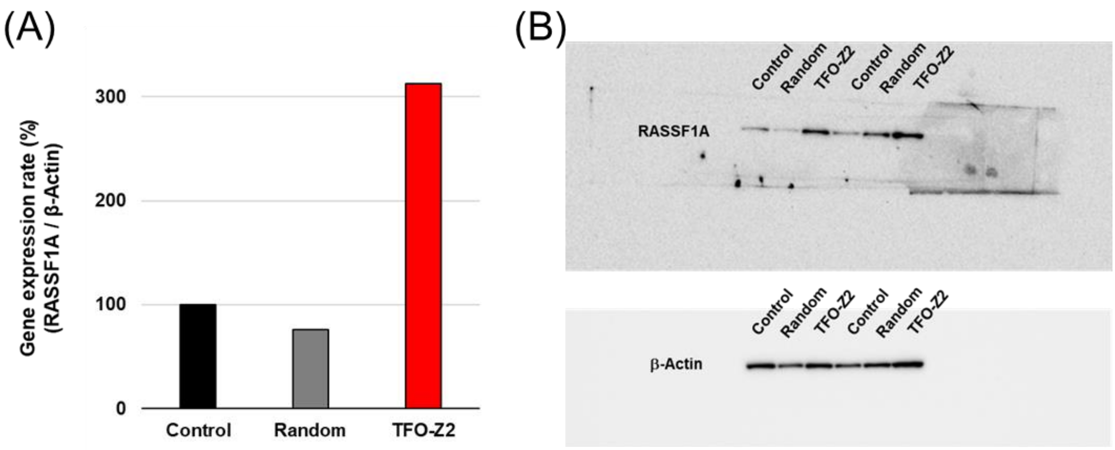

Figure S7 Activation of RASSF1A gene expression in MCF-7 cells transfected with TFOs. MCF-7 cells ($8{\times}10^4$ cells/well) were transfected with TFOs (Random, TFO-Z2; 200 nM) and Lipofectamine 2000 for 3 hrs and then incubated at 37°C for 24 hrs. (A) The expression rate of RASSF1A was normalized with the expression of β-actin measured by Western blotting in Figure 2C. (B) The full raw data of Western Blot experiment in Figure 2C.

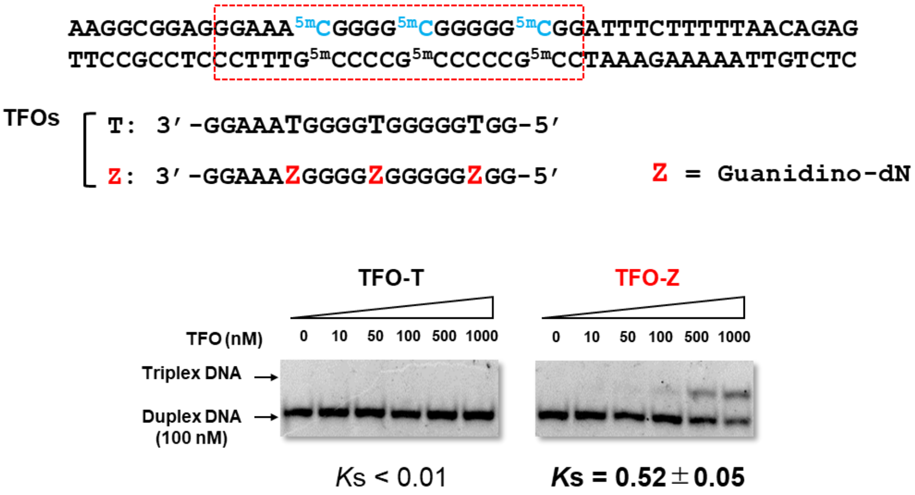

Figure S8 The target sequence of the p16 promoter and TFOs (TFO-T and TFO-Z) forming triplex DNA for the target sequence. FAM-labeled p16 duplexes (25 bp; 100 nM) were incubated with increasing concentrations of each TFO (19-mer; 0–1000 nM) in buffer containing 20 mM Tris-HCl and 2.5 mM $MgCl_2$ at 37°C and pH 7.5. Electrophoresis was performed with a 10% non-denaturing polyacrylamide gel at 4°C.

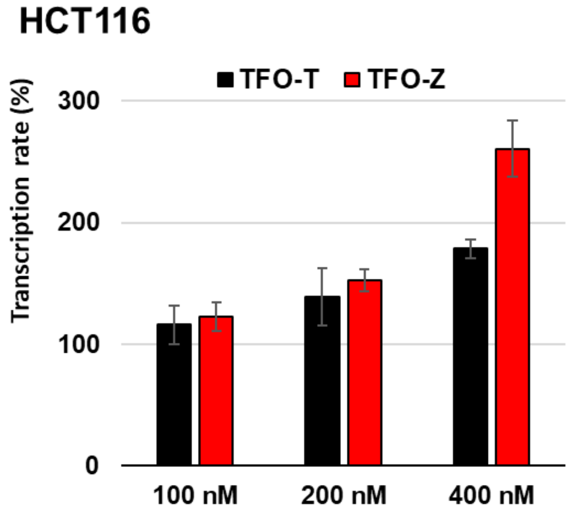

Figure S9 Effect of p16 gene transcription in HCT116 cells (2×10^4 cells/well) transfected with TFOs (100-400 nM) and Lipofectamine 2000 for 3 hrs and incubated at 37°C for 24 hrs. The transcription rate was calculated based on the *ΔΔ*Ct method by correcting the transcripts of p16 normalized with that of GAPDH measured by qRT-PCR.

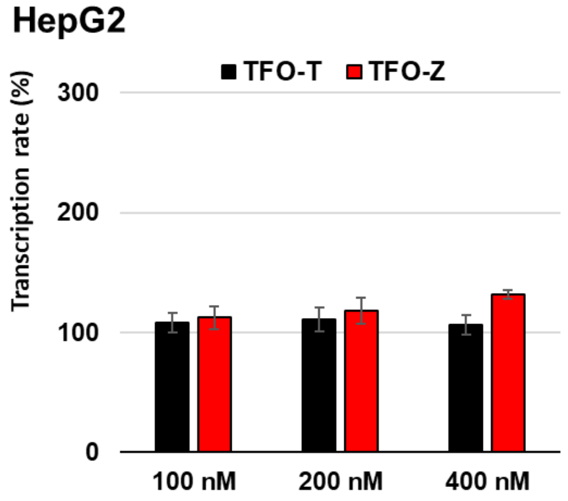

Figure S10 Effect of p16 gene transcription in HepG2 cells (2×10^4 cells/well) transfected with TFOs (100-400 nM) and Lipofectamine 2000 for 3 hrs and incubated at 37°C for 24 hrs. The transcription rate was calculated based on the *ΔΔ*Ct method by correcting the transcripts of p16 normalized with that of GAPDH measured by qRT-PCR.
